# Efficacy of Hypozalix spray and propolis mouthwash for prevention of chemotherapy-induced oral mucositis in leukemic patients: A double-blind randomized clinical trial

**DOI:** 10.15171/joddd.2016.036

**Published:** 2016-12-21

**Authors:** Hosein Eslami, Firouz Pouralibaba, Parisa Falsafi, Sepideh Bohluli, Babak Najati, Ramin Negahdari, Milad Ghanizadeh

**Affiliations:** ^1^Department of Oral Medicine, Faculty of Dentistry, Tabriz University of Medical Sciences, Tabriz, Iran; ^2^Orthodontics Research Center, Department of Orthodontics, School of Dentistry, Shiraz University of Medical Sciences, Shiraz, Iran; ^3^Department of Prosthodontics, Faculty of Dentistry, Tabriz University of Medical Sciences, Tabriz, Iran; ^4^Department of Oral and Maxillofacial Surgery, Faculty of Dentistry, Tabriz University of Medical Sciences, Tabriz, Iran

**Keywords:** Artificial saliva, leukemia, mucositis, propolis, chemotherapy

## Abstract

***Background.*** Oral mucositis is the chief complication of head and neck chemotherapy. This study was conducted to evaluate Hypozalix artificial saliva and propolis mouthwash efficacy for the prevention of chemotherapy-induced oral mucositis in leukemic patients.

***Methods.*** The present double-blind clinical trial was carried out on 72 patients undergoing chemotherapy. The patients were assigned to 3 groups. In the control group, CHX mouthwash and fluconazole were used by the subjects. In groups 1 and 2, Hypozalix and propolis mouthwashes were added to the combination therapy used in the control group. The results were compared between the three groups after 14 days.

***Results.*** Mean score A was significantly higher than mean score B in children (P = 0.001). In contrast, mean score A was significantly lower than mean score B in young adults (P = 0.003).

***Conclusion.*** Use of Hypozalix spray or propolis mouthwash in association with CHX mouthwash and fluconazole simultaneously at the start of chemotherapy resulted in a decrease in chemotherapy complications after 14 days. In many cases the use of propolis mouthwash yielded better results and the patients exhibited a greater tendency to continue to use it.

## Introduction

Stomatitis or oral mucositis is a term used to describe inflammation of the oral mucosa as a general and debilitating complication caused by chemotherapy and radiotherapy of the head and neck region.^1^ From a clinical standpoint, the effects of chemotherapy on oral mucosa start after a short time, reach a peak in 7‒10 days and continue for two weeks.^2,3^ In a study, the incidence of oral mucositis in children with cancer was reported to be 52‒81%.^3^ Stomatitis is a common finding in a way that 10% of patients receiving adjuvant chemotherapy, 40% of patients receiving induction chemotherapy, 80% of patients treated for stem cell transplantation and 100% of patients receiving radiotherapy of the head and neck region suffer from this complication.^4,5^ Stomatitis begins with a mild redness (erythema) and may develop toward edema, painful sores, hemorrhage and even localized or systemic infections.^1,6^ Patients with severe oral mucositis sometimes cannot even eat, speak or swallow due to pain.^7^ The wounds caused by oral mucositis may become infected as a result of opportunistic infections (viruses, fungi and bacteria) due to the decreased ability of the immune system as a result of chemotherapy, complicating the process of diagnosis and treatment.^3^ Lesions in mucositis caused by chemotherapy are usually seen in non-keratinized mucosa such as the lateral and ventral surfaces of the buccal mucosa, tongue and soft palate, which will double the patient's nutritional problems.^3,4,7^

Although there is still no definite method for the treatment and prevention of oral mucositis, various measures, including oral and dental hygiene,^8^ different mouthwashes,^9-11^ topical anesthetics such as lidocaine,^12^ diphenhydramine,^13^ nystatin, sucralfate and psychotherapy^12^ are currently suggested. In addition, given many complications of stomatitis caused by chemotherapy, its prevention significantly reduces the cost of health care and also increases patient survival, and use of propolis and artificial saliva is among these methods.^14^

Propolis is a substance that is used for the treatment of oral mucositis.^6^ Propolis is a natural product derived from plant resin collected by bees, which is mixed with the salivary enzymes of bees. Bees use it to repair the walls of the hive and to protect their colony from diseases.^15,16^ Synthetic propolis which has been mixed in 70‒90% ethyl alcohol has been used traditionally to treat oral ulcers, denture-induced stomatitis, aphthous stomatitis and microbial and fungal infections.^17,18^ Propolis has properties such as stimulating cellular and humoral immune systems, anesthesia and analgesia, and anti-oxidant and anti-inflammatory properties; it strengthens soft connective tissue and inhibits the activity of some hydrolases, oxidoreductases and kinases.^1,6,17-20^ The effective role of propolis has been shown in the treatment of periodontal and oral infections. Moreover, radiotherapists have successfully used propolis in the treatment of stomatitis and mucositis.^11,21,22^

Abdulrahman et al strongly recommended use of honey and its derivatives such as propolis because of acceleration of the repair process of oral mucositis caused by chemotherapy. Their reason for using propolis was its antimicrobial properties and anti-oxidative and anti-ulcer and anti-tumor lesion properties.^6^

It was observed in a study by Benderli et al that propolis can be used in mice as a reducing agent for severe mucositis caused by radiation of the head and neck region.^19^ However, the results in another study on the effect of propolis in the treatment of severe oral mucositis in children undergoing chemotherapy showed no significant differences between placebo and propolis and according to this study, propolis was not recommend in the treatment of severe mucositis.^1^

A decrease in salivary flow rate is another factor increasing the risk of oral mucositis and the use of artificial saliva can result in a normal oral flora, create lubrication properties in the oral cavity and has the ability to heal wounds.^23^ It was observed in a study on the role of artificial saliva in decreasing the side effects of radiotherapy of the head and neck region; furthermore, artificial saliva was more effective in the treatment of patients with oral mucositis with a reliability rate of more than 99% compared to placebo.^23^

On the other hand, Vissink et al suggested that different types of carboxymethyl cellulose- or mucin-based artificial saliva had no effect on decreasing the risk of inflammation and infection of the oral cavity despite having a role in the comfort of patients with salivary gland function disorders.^24^

Given that usual medical treatments have limited efficacy in improving oral mucositis, alternative treatments are very important for its prevention and control. Based on various studies, there is controversy regarding the effectiveness of propolis and artificial saliva in the prevention of chemotherapy-induced oral mucositis. Thus, this study aimed to compare the effectiveness of these two alternative methods.

## Methods

In this double-blind clinical trial, 72 patients undergoing chemotherapy, with an age range of 18‒71 years, referring to the Oncology Department of Shahid Ghazi Hospital in Tabriz, were evaluated. All the patients were initially examined and the study procedures and the advantages and complications were explained to the patients. All the patients were matched in relation to age, sex and the regimen of chemotherapy (medication and dose). Written informed consent was taken from the patients who were willing to participate in the study and then they were included in the study. The exclusion criteria were (a) allergy to propolis, chlorhexidine (CHX), fluconazole and Hypozalix; (b) pre-diagnosed oral diseases or therapy for oral diseases; (c) systemic diseases other than malignancy such as diabetes mellitus, hypertension, autoimmune diseases, renal failure, and graft-versus-host disease; and (d) use of medications which can reduce salivary flow.

The tool used to collect data was a two-part questionnaire and a checklist to determine the severity of mucositis. The two-part questionnaire was used to collect demographic data (age and gender) and consisted of questions on the type of disease, history of chemotherapy, presence or absence of systemic disease other than malignancy, and the presence or absence of skin or respiratory allergy. The second part of the questionnaire consisted of questions on the severity of xerostomia, ease of mastication and swallowing, the severity of burning sensation, the quality of sleep during the night and the tendency to continue to use the medications; the patients completed this part before and after intervention. In addition, in order to determine the severity of oral mucositis a checklist was used, which was designed based on the criteria of WHO; based on these criteria, oral mucositis is divided into 5 distinct grades from 0 to 4.^17^To determine the validity of the questionnaire, content validity method was used. In addition, in order to determine the reliability of the questionnaire, simultaneous observation technique was used. To this end, observations were carried out by two observers with similar characteristics, using similar guidelines on 10 samples, which yielded a correlation coefficient of 0.94. Then the questionnaire was completed using interviews and patient files.

### Determination of the sample size

The formula for calculating the sample size was used based on a similar study to compare two means^1^ and based on the following formula, at least 24 patients were enrolled in each group and the total number of patients was calculated at 72 individuals.

$$\frac{\left(z1-\frac{\propto }{2}+z1-\frac{\beta }{2}\right)\times p1\left(1-p1\right)\times p2\left(1-p2\right)}{{\left(p1-p2\right)}^{2}}$$

$$\text{α}=\frac{0}{05}\;z_{\left(1-\frac{\alpha }{2}\right)}=\frac{1}{96}\;\;\;\;z_{\left(1-\beta \right)}=\frac{1}{28}$$

One oncologist used the Randlist software program to randomly assign all the patients to three equal groups.

Simultaneously at the start of chemotherapy, all the patients began to rinse their oral cavities with mouthwashes. The first group received chlorhexidine (CHX) mouthwash and fluconazole with Hypozalix artificial saliva (Biocodex, France), containing 100 mL of the solution with approximately 200 puffs and the second group received CHX mouthwash and fluconazole with propolis mouthwash and the third group (control group) received CHX mouthwash and fluconazole. Hypozalix artificial saliva was used according to manufacturer's instructions: 4 to 5 puffs every 8 hours and each time on the left or right buccal mucosa and the second group used propolis mouthwash for 14 days, every day three times by gargling 10 mL of the mouthwash in the mouth for one minute each time and then spitting out. The results were compared between the three groups after 14 days. All the subjects completed the study and none was excluded from the study.

In this double-blind study, examination and selection of patients and prescription of mouthwashes were carried out by one oncology specialist and the drugs were delivered by a nurse and periodic examinations were carried out after using the drugs by a postgraduate student of oral medicine under the supervision of an oral medicine specialist. Both of them were blinded to the type of the prescribed agents.

This study was registered in the Center of Clinical Trials under the code IRCT 139311204385N1 and the ethical code of 93148. This randomized controlled clinical trial was carried out from July 2014 to December 2014.

### Statistical analysis

Data were analyzed with descriptive statistics (frequencies, percentages, means or medians) using SPSS 16. Statistical significance was set at P < 0.05.

### Ethics approval

All the ethical and the humanity considerations were observed and performed according to the Helsinki Declaration of 1975, as revised in 2008. All the human experiments were approved by the Ethics Committee of the Tabriz University of Medical Sciences. Detailed informed consent form was obtained from all the participants.

## Results

### Xerostomia

The results of the present study showed that after 14 days, 50% of the patients in the control group, who only used CHX mouthwash and fluconazole, exhibited signs of recovery from xerostomia. In group 2, in which the patients used propolis mouthwash in addition to CHX mouthwash and fluconazole, 50% of patients exhibited some signs of recovery from xerostomia. However, 95.8% of patients in group 1, who used CHX mouthwash and fluconazole and Hypozalix spray, reported recovery from xerostomia. Chi-squared test showed that the difference was statistically significant (P = 0.0006).

### Chewing and swallowing

At the end of the study, 29.17% of the patients in the control group, 87.5% in group 1 and 70.83% in group 2 exhibited easy mastication. The highest rate of difficulty in mastication was seen in the control group, followed by group 2. Chi-squared test showed that the difference was significant statistically (P = 0.0001). In addition, 33.3% of the patients in the control group, 63.3% of the patients in group 1 and 87.5% of the patients in group 2 exhibited easy swallowing. The greatest difficulty in swallowing was seen in the control group, followed by group 1. Chi-squared test showed that this difference was statistically significant (P < 0.0001).

### Tendency to continue to use the product 

At the end of the study, 33.3% of the patients in the control group, 58.3% in group 1 and 91.6% in group 2 were interested in continuing to use the product. The lowest tendency was seen in patients of control group, followed by those in group 1. Based on chi-squared test the difference was statistically significant (P = 0.0002).

### Cessation of waking up and a decrease in burning sensation and feeling comfortable with the product

In addition, 16.67% of patients in the control group, 25% in group 1 and 62.5% in group 2 exhibited more full night’s sleep and less waking up. Chi-squared test revealed that the difference was statistically significant (P = 0.0018).

In relation to an improvement in oral burning sensation, 8.33% of the patients in the control group, 25% in group 1 and 91.67% in group 2 exhibited a decrease in burning sensation. The greatest burning sensation was detected in control group patients, followed by those in group 1. Chi-squared test showed that the difference was significant statistically (P < 0.0001).

Moreover, 62.5% of the patients in the control group, 62.5% in group 1 and 95.8% in group 2 had a higher rate of feeling comfortable. Chi-squared test showed that the difference was significant (P = 0.0103).

### Severity of mucositis

**Figure 1.**
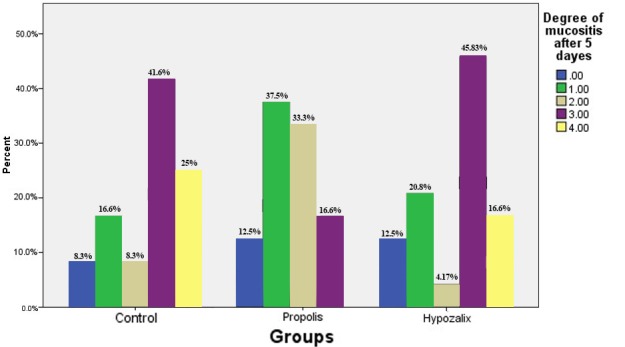
Evaluation of mucositis grades 5 days after using artificial saliva and propolis mouthwash.

A clinical examination of patients carried out 5 days after using the medications showed that 8.33% of patients in the control group, 12.5% in group 1 and 50% in group 2 were free of mucositis. However, 25% of patients in the control group and 16.6% in group 1 had grade 4 mucositis. None of the patients in group 2 exhibited grade 4 mucositis (Figure 1). Chi-squared test showed that the difference was significant (P = 0.0007).

**Figure 2.**
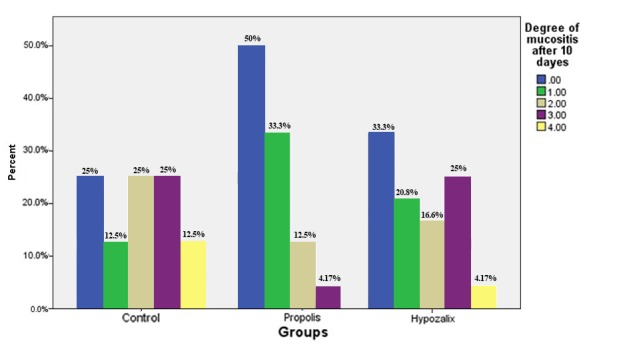
Evaluation of mucositis graded 10 days after using artificial saliva and propolis mouthwash.

A clinical examination of patients carried out 10 days after using the medications showed that 25% of the patients in the control group, 33.3% in group 1 and 50% in group 2 were free of mucositis. In addition, 12.5% of the patients in the control group and 4.17% in group 1 had grade 4 mucositis. None of the patients in group 2 had grade 4mucositis (Figure 2). However, chi-squared test indicated that the difference was not statistically significant (P = 0.1135).

## Discussion

Statistics show that 40% of adults and 90% of children under 12 years of age, who undergo chemotherapy, suffer from side effects of chemotherapy. The most common complications are mucositis (inflammation of the lining of the oral cavity), gingival infections, candidiasis and oral ulcers that can also cause septicemia with oral origin.^1^ According to a study by Anthony et al, oral complications can be seen in 89% of patients undergoing chemotherapy.^25^ Despite the fact that no definite method has been identified for the treatment and prevention of oral mucositis, several measures that significantly reduce the cost of health care and increase patient survival have been proposed, and use of propolis and artificial saliva are among these methods. The present study was conducted to evaluate the efficacy of Hypozalix artificial saliva and propolis mouthwash in the prevention of chemotherapy-induced oral mucositis in leukemic patients.

The results revealed that 50% of the patients in the control group, 95.8% of the patients who received Hypozalix and 50% of those who received Propolis exhibited a decrease in xerostomia severity. Since xerostomia affects the chewing and swallowing functions,^7^ we tried to evaluate the effectiveness of these drugs in chewing and swallowing in this study. At the end of the study, 29.17% of the patients in the control group, 87.5% in the Hypozalix group and 70.83% in the Propolis group exhibited easy mastication. In addition, 33.3% of the patients in the control group, 63.3% of the patients in the Hypozalix group and 87.5% of the patients in the Propolis group exhibited easy swallowing.

A decrease in salivary flow rate is another factor that increased the risk of oral mucositis. However, artificial saliva can result in a normal oral flora, create lubrication properties in the oral cavity and help heal wounds,^23^ as shown in a study on the role of artificial saliva in reducing the side effects of radiotherapy of the head and neck. On the other hand, artificial saliva was more effective in the treatment of patients suffering from oral mucositis with a reliability rate of more than 99% compared to placebo.^23^

Dirix et al reported that 93% of patients with head and neck cancer after radiotherapy suffer from dry mouth and 65% of them have moderate to severe xerostomia.^26^

Noronha et al assessed the preventive effect of a mucoadhesive gel having 5% of Brazilian green propolis for radiation-induced oral mucositis. All the patients reported that the sensation of "dry mouth" was not observed during the intervention. This probably occurred because propolis has acidic contents that could contribute to salivary flow.^27^

In an animal study, Motallebnejad et al showed that propolis was beneficial in decreasing radiation-induced xerostomia and might be useful for head and neck cancer patients.^28^

Ameri et al compared the efficacy of a herbal compound containing *Malva sylvestris* and *Alcea digitata* (Boiss) with artificial saliva (Hypozalix) for improving the symptoms of xerostomia in head and neck cancer patients and reported that in the herbal group there was a significant difference between the grades of dry mouth before and after the intervention, but no changes were detected in the grades of dry mouth in the Hypozalix group.^29^

Seema Devi et al reported lack of stability in the oral cavity of patients after radiation therapy. They also showed the need for dental care during and after radiation therapy.^30^

According to a study by Jelmma et al, Xialine artificial saliva improved the quality of life and the senses of taste and smell. These researchers also demonstrated a positive effect on speaking.^10^

In addition, 91.6% of the patients in the propolis group were interested in continuing to use the product. The lowest tendency was seen in the control group patients, followed by those in the Hypozalix group.

A total of 62.5% of the patients in the propolis group exhibited less waking up at night and 95.8% of them had a higher rate of feeling comfortable. Also, the lowest burning sensation was detected in that group.

In addition, propolis resulted in a significantly greater decrease in the severity of mucositis compared to Hypozalix and control group 5 days after using the medications.

Tomazevic and Jazbec showed that nearly half of the children undergoing chemotherapy enrolled in their study (42% and 48% of patients in the propolis and placebo groups, respectively), suffered from severe oral mucositis (OM). It was also shown that severe OM was of slightly shorter duration and of a lower extent in the propolis group.^1^

Suemaru et al indicated that 0.3%, 1% and 3% propolis had no positive effect on 5-fluorouracil-induced oral mucositis in hamsters,^31^ but Motallebnejad et al reported that an increasing dose of Iranian propolis could decrease the severity of radiotherapy-induced mucositis in rats.^21^

These contradictory results might be attributed to differences in study designs and also to differences in propolis origins.

Abedipour et al evaluated the effects of chlorhexidine and Persica mouthwashes containing *A. Millefolium* on prevention of stomatitis in patients undergoing chemotherapy and reported that both mouthwashes had similar properties.^32^

In the present double-blind study, all the samples were selected randomly and equally; therefore, the odds of bias was minimized. In many cases use of propolis mouthwash yielded significantly better results, and the patients exhibited a greater tendency to continue to use it.

If the results of this study are confirmed by other studies, treatment with Propolis can reduce secondary oral infections and mucositis caused by chemotherapy.

Since this medication is available in Iran, is low in price, has acceptable flavor and smell, is easy to use, is non-invasive and is considered a non-chemical agent with no side effects, it will be more favorably accepted by Iranian patients compared to other medications.

During this study, the researchers encountered some problems, including the difficulty of finding an adequate number of samples, the following of patients who were in a critical condition and lack of cooperation on behalf of the patients; these problems were overcome by allocating sufficient time for the study. In addition, since Hypozalix has no official agency and dispenser in Iran, it was difficult to provide the spray for the study. Since Propolis and Hypozalix had positive effects on decreasing mucositis induced by chemotherapy in the present study, it is suggested that toothpastes be designed with propolis base so that patients can use them on a daily basis.

## Conclusion

Based on the results of the present study, Hypozalix spray or propolis mouthwash in association with CHX mouthwash and fluconazole simultaneously at the start of chemotherapy resulted in a decrease in chemotherapy complications after 14 days. Furthermore, propolis mouthwash yielded better results and the patients exhibited a greater tendency to continue to use it.

## Acknowledgements

The authors would like to thank Tabriz University of Medical Sciences for financial support of the study.

## Authors’ contributions

HE and RN were responsible for the design and concept of the study as well as revision of the prepared manuscript. PF, RN and SB analyzed the data, carried out the literature search and drafted the manuscript. BN, HE and MGH performed the clinical evaluations. All the authors have read and approved the final manuscript.

## Funding

This research was financially supported by Tabriz University of Medical Sciences (Ref No: 203/T).

## Competing interests

The authors declare that they have no competing interests with regards to authorship and/or publication of this paper.

## Ethical approval

This study was approved by the Ethics Committee of Tabriz University of Medical Sciences in Iran and Iranian Registry of Clinical Trials (IRCT139311204385N1).
